# Non-antibiotic antimicrobial agents for chronic rhinosinusitis: a narrative review

**DOI:** 10.1016/j.bjorl.2024.101436

**Published:** 2024-04-26

**Authors:** Joao Vitor Bizinoto Caetano, Fabiana Cardoso Pereira Valera, Wilma T. Anselmo-Lima, Edwin Tamashiro

**Affiliations:** Universidade de São Paulo, Faculdade de Medicina de Ribeirão Preto, Departamento de Oftalmologia, Otorrinolaringologia e Cirurgia de Cabeça e Pescoço, Ribeirão Preto, SP, Brazil

**Keywords:** Chronic rhinosinusitis (CRS), Bacterial infection, Non-antibiotic, Antibiofilm agents, Topical therapies

## Abstract

**Objective:**

This narrative review explores alternative non-antibiotic antimicrobial agents for CRS management in adults.

**Methods:**

Alternative antimicrobial agents using EPOS 2020 guidelines as reference were selected, and articles dated from 2003 to 2022 in English, Portuguese, or Spanish using PubMed and EMBASE databases. The parameters analyzed included study design, evidence level, population characteristics, CRS characteristics, interventions, outcomes, sample size, randomization, blinding, and side effects. Reviews, unrelated contexts,in vitro experiments, and duplicates were excluded.

**Results:**

148 articles were screened; 19 articles were selected for analysis. Randomized controlled trials and cohort studies assessing non-antibiotic antimicrobial treatments for CRS were included. Xylitol demonstrated effectiveness in reducing CRS symptoms, particularly SNOT-22 scores, surpassing saline irrigation benefits. Manuka honey showed potential microbiological benefits in recalcitrant CRS, but symptomatic and endoscopic improvements remained inconclusive. Baby shampoo irrigation improved nasal mucociliary clearance and postoperative outcomes. Colloidal silver nasal irrigation showed limited efficacy in reducing CRS symptoms or endoscopic scores. Povidone-Iodine (PI) nasal irrigation yielded mixed results, with varying effects on culture negativity and SNOT-20 scores. Bacteriophage treatment exhibited promise in decreasing specific bacterial strains and cytokine levels.

**Conclusion:**

Non-antibiotic antimicrobial therapies, including xylitol, manuka honey, baby shampoo, colloidal silver, PI, bacteriophages, lactoferrin, and carrageenan offer potential alternatives for CRS in adult patients. Xylitol, baby shampoo, and PI presented benefits in improving symptoms and nasal endoscopic scores, however, the number of studies is limited for conclusive recommendations and safety assessments. CRS management should adopt a comprehensive approach, particularly for non-infectious or immune-related cases, moving beyond antibiotics. Antibiotics should be reserved for confirmed bacterial infections. Overall, this review shows the importance of exploring non-antibiotic therapies to enhance the management of CRS.

## Highlights

• Non-antibiotic antimicrobial therapies offer potential alternatives in CRS patients.
• Most non-antibiotic antimicrobial therapies did not show important side effects.
• The majority of studies reviewed have low level of evidence.

## Introduction

Chronic Rhinosinusitis (CRS) is a common inflammatory condition that affects the nose and sinuses in about 5% of the population, causing symptoms such as nasal congestion, nasal secretion, facial pain, or smell loss.^1^ By definition, chronic rhinosinusitis is any inflammatory condition of the nose and paranasal sinuses that causes sinonasal symptoms (the presence of rhinorrhea or nasal congestion/obstruction is mandatory) for at least 12 weeks, confirmed by inflammatory alterations in nasal endoscopy or computed tomography.^2^

Currently, the majority of primary CRS is considered non-infectious diseases, as evidence has emerged suggesting that antibiotics may not be effective in treating CRS and may even be harmful in some cases.^3^, ^4^ However, bacteria may play a role in part of patients with CRS, as bacterial biofilms colonizing the upper respiratory tract are relatively common in these patients and influence the inflammatory responses by the host.^5^, ^6^ Antibiotic resistance may arise from the biofilm structure and molecules involved in the bacterial cell-cell communication (quorum sensing), which relies on the extracellular concentration of bacterial byproducts that ultimately regulate gene expression of several virulence and phenotypic changes in the bacterial form of living.^7^ Understanding the biofilm biology and their antibiotic resistance is crucial for the development of new treatment approaches.

Measures to avoid pathogenic bacterial colonization, biofilm formation, or even biofilm eradication are challenging in the clinical setting, as biofilms present high resistance to innate and adaptive responses and especially to antibiotics.^8^ For instance, one of the main difficulties in treating CRS with antibiotics is that it is often caused by factors other than bacteria, such as allergies, environmental irritants or underlying immune system disorders. In these cases, antibiotics will have little to no effect on the underlying inflammation and may even contribute to the development of resistant bacteria.

Treating CRS with antibiotics poses side effect risks, ranging from mild gastrointestinal symptoms to severe complications. Prolonged use may lead to superinfections like *Clostridium difficile* colitis. Antibiotics may be suitable for bacterial CRS or post-surgery cases but are generally ineffective in improving symptoms or endoscopic aspects in most CRS cases.^4^, ^9^, ^10^ Antibiofilm therapy may alleviate inflammation in CRS by targeting biofilm colonization, reducing acute exacerbations.^10^, ^11^ To minimize antibiotic side effects in CRS, alternative antimicrobial/antibiofilm therapies, supported by in vitro and preclinical studies, have been explored.

In this study, we reviewed the current evidence of several non-antibiotic treatments with antibacterial effects on CRS patients, including xylitol, manuka honey, baby shampoo, colloidal silver, Povidone-Iodine (PI) and bacteriophage as potential options for treating CRS.

## Objectives

To review the current evidence of clinical effects of non-antibiotic agents with antibacterial activity for the treatment of CRS. Several agents tested in CRS patients are highlighted, including their mechanisms of action, efficacy, and toxicity.

## Methods

The selection of relevant agents was based on EPOS 2020,^2^ including articles in English, Portuguese, or Spanish. The publications considered ranged from 2003 to 2022, and the databases used for research were Pubmed and EMBASE. The Mesh terms employed were: “xylitol” AND “sinusitis” OR “xylitol” AND “rhinosinusitis”; “baby shampoo” AND “sinusitis” OR “baby shampoo” AND “rhinosinusitis”; “colloidal silver” AND “sinusitis” OR “colloidal silver” AND “rhinosinusitis”; “bacteriophage” AND “sinusitis” OR “bacteriophage” AND “rhinosinusitis”; “manuka honey” AND “sinusitis” OR “manuka honey” AND “rhinosinusitis”; “Povidone-iodine OR PVPI” AND “sinusitis” OR “Povidone-iodine OR PVPI” AND “rhinosinusitis”.

For selected articles the following parameters were analyzed: study design, level of evidence, population characteristics, CRS phenotype/endotype, comorbidities, intervention methods, outcomes, sample size, randomization, blinding, and side effects. All articles apart from clinical trials were excluded from this review.

Two researchers (JVBC and ET) independently selected articles. Findings were compiled into a table to decide inclusion. Exclusion criteria were review articles, corrigendum, annuaries, unrelated contexts, in vitro experiments, and duplicates. Discrepancies in article selection were resolved through author discussions.

## Results

**Figure 1:**
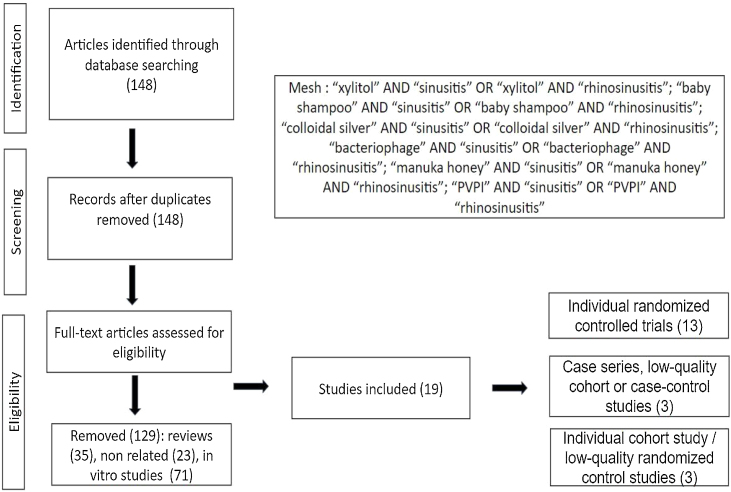
Flowchart of study selection.

Out of 148 articles meeting the criteria, 129 were excluded (71 in vitro studies, 35 reviews, 23 unrelated to CRS), leaving 19 for analysis. These included 13 randomized controlled trials, 3 case-series/low-quality cohort/case-control studies, and 3 cohort study/low-quality randomized controlled studies (Fig. 1). None of the studies included pediatric patients, under 18-years-old. Data are summarized in Table 1, Table 2, Table 3.

**Table 1:** Summary of study design.

Agent	Authors	Type of study	Evid level	Population	Phenotype/Endotype
	Rabago et al., 2020	Individual randomized controlled trials	1b	53.8 ± 7.8 years	CRS mixed
	Silva et al., 2022	Individual randomized controlled trials	2b	18+ years	CRS mixed, difficult-to- treat CRS
Xylitol	Weissman et al., 2011	Individual randomized controlled trials	1b	18+ years	CRS mixed, post-surgery
	Lin et al., 2017	Individual randomized controlled trials	1b	35‒67 years	CRS mixed, post-surgery
	Kim et al., 2019	Individual randomized controlled trials	1b	26‒56 years	CRS mixed
	Thamboo et al., 2011	Individual randomized controlled trials	1b	19+ years	Allergic Chronic Rhinosinusitis
	Ooi et al., 2019	Individual randomized controlled trials	1b	27‒86 years	CRS mixed, post-surgery
Manuka Honey	Lee et al., 2017	Individual randomized controlled trials	1b	51‒64 years	CRS mixed, post-surgery
	Chang et al., 2011	Individual randomized controlled trials	1b	17‒80 years	CRS mixed
	Wong et al., 2011	Case series, low-quality cohort or case- control studies	4	31 and 71-years-old	Allergic Fungal Rhinosinusitis
	Tulaci et al., 2021	Case series, low-quality cohort or case-control studies	4	18‒65 years	CRSwNP
Baby Shampoo	Chiu et al., 2008	Individual cohort study/low-quality randomized control studies	4	Not informed	CRS mixed
	Farag et al., 2013	Individual randomized controlled trials	2b	18+ years	CRS mixed, post-surgery
Colloidal Silver	Scott et al., 2017	Individual randomized controlled trials	1b	44−86 years	Recalcitrant CRS, post-surgery
	Panchmatia et al., 2019	Individual cohort study/low-quality randomized control studies	2b	63 ± 14 years	CRS mixed
Povidone-Iodine (PI)	Wu et al., 2022	Individual randomized controlled trials (with narrow confidence intervals)	1b	45 ± 13 and 39 ± 13 years	CRS + hipertrophic inferior turbinate, post-surgery
	Lee et al., 2020	Individual cohort study/low-quality randomized control studies	2b	36‒52 years	CRS mixed
	Dobretsov et al., 2021	Individual randomized controlled trials	1b	18‒64 years	CRSwNP, post-surgery
Bacteriophages	Rodrigues et al., 2022	Case series, low-quality cohort or case-control studies	4	61 years-old	CRSsNP

Agent	Authors	Comorbities	Intervention	*n* =	Random	Bliding
	Rabago et al., 2020	Smoking	Three Groups:	40	Yes	Yes
			1- Standard treatment for CRS			
			2- PT +120 mL saline 2% 2× day (Netipot) 3- TP +120 mL 5% xylitol 2× a day (Netipot)			
	Silva et al., 2022	N/A	Xylitol 6 g/360 mL saline (1.7%), 60 mL 3×/day for 30 days vs. Saline, 60 mL 3×/day for 30 days	52	N/A	N/A
Xylitol	Weissman et al., 2011	None	Cross-over Trial.	20	Yes	Yes
			10 days of treatment, with 3 days of washout before switching.			
			Xylitol 5% 240 mL 1× day 10 days vs. Saline 240 mL 1× day, 10 days.			
	Lin et al., 2017	None	Xylitol 5% 240 mL 1× day 30 days vs. Saline 240 mL 1× day, 30 days.	25	Yes	Yes
	Kim et al., 2019	None	Cross-over trial	100	Yes	Yes
			1- Xylitol 1.7% in saline 0.8% in 240 mL, 3× a day for 14 days.			
			2- Saline 0.9 mL, 240 mL, 3× a day for 14 days. Seven days washout between treatments. All received topical nasal steroids post-op.			
	Thamboo et al., 2011	N/A	One nostril spray of 2 mL of 50/50 honey-saline, 1× a day for 30 days	38	Yes	Yes
	Ooi et al., 2019	Asthma, NSAID sensitivity	16.5% MH +1.3 mg/mL MGO sinonasal rinses and concurrent 10 days of placebo tablet vs. 14 days of twice-daily saline sinonasal rinses and concurrent 10 days of culture-directed antibiotic therapy.	25	Yes	Yes
	Lee et al., 2017	Asthma, allergies, and NSAID sensitivity	10% Manuka half bottle 2× day (Medihoney) vs saline for 30 days.	42	Yes	Yes
			Both groups received appropriate antibiotics.			
Manuka Honey	Chang et al., 2011	Not Informed	Three groups on one operated side, applied in the intraop:	48	Yes	Yes
			- Merocel soaked with budesonide on one side.			
			- Merocel soaked with genta on one side.			
			- Merocel soaked with manuka on one side.			
			The contralateral side received merocel, as control.			
	Wong et al., 2011	Asthma	Two cases of allergic fungal rhinosinusitis treated with conventional treatment + manuka honey 120 mL 2 times per day, before and after 12 weeks.	2	No	No
	Tulaci et al., 2021	None	Baby shampoo 1%, 240 mL, 4×/day for 4 weeks.	77	No	No
Baby Shampoo	Chiu et al., 2008	N/A	Baby shampoo 1%, 60 mL each nostril, 2×/day for 4 weeks.	18	No	No
	Farag et al., 2013	N/A	Baby shampoo 1%, 240 mL 3×/day vs. Homemade hypertonic solution 240 mL 3×/day	40	Yes	No
Colloidal Silver	Scott et al., 2017	Asthma, smoking	Everyone treated with antibiotics.	22	Yes	Yes
			Nasal wash with colloidal silver 0.015 mg/mL, 240 mL, 2×/day for 10 days vs. Saline 0.9%, 240 mL, 2×/day for 10 days.			
	Panchmatia et al., 2019	N/A	PVPI 0.08% (2 mL PVPI 10% in 240 mL SF) 3× week, 1× day, for 7 weeks, plus Budesonide 1% 240 mL.	29	No	No
Povidone-Iodine (PI)	Wu et al., 2022	Asthma, smoking	PVPI 1% vs saline (240 mL 2× day)	57	Yes	yes
			PVPI 0.1% vs. 0.05% mupirocine vs. saline.			
	Lee et al., 2020	Asthma, smoking	All received antibiotics when appropriate, oral corticosteroids when needed, and/or high-volume nasal irrigation with budesonide			
				65	Yes	Yes
	Dobretsov et al., 2021	N/A	Othopag vs Placebo. Applied below middle turbinate 2×/day for 10 weeks after surgery.	40	Yes	Yes
Bacteriophages	Rodrigues et al., 2022	Diabetes, sarcoidosis	Specific phage therapy + antibiotics.	1	No	No

**Table 2:** Summary of outcomes and results.

Agent	Authors	Outcomes	Summary of results
Xylitol	Rabago et al., 2020	SNOT-20, SF-36, Mutidimension fatigue index (MFI), 8- and 26-weeks following treatment.	Xylitol improved SNOT-20 from baseline at 8 and 26 weeks, but only at 26 weeks it was better than control (standard care). 2% saline improved SNOT-20 from baseline only at 26 weeks, with no differences from control at 8 and 26 weeks.
	Silva et al., 2022	NOSE, SNOT-22, VAS for pain	The Xylitol group showed an average reduction of 32.9 points in SNOT-22, greater than the reduction of the “Saline solution” group (average of 21.3 points).
	Weissman et al., 2011	SNOT-20, VAS for general symptoms	Saline increased SNOT-20 by 3.93 points; xylitol decreased by 2.43 points (significant difference). Saline decreased VAS general symptoms by 0.07; xylitol increased by 0.56 (no significant difference).
	Lin et al., 2017	SNOT-22, VAS for general symptoms iNOS mRNA, nasal NO right max sinus	Xylitol Improved VAS, reduced SNOT-22, increased nasal NO concentration and right maxillary sinus iNOS expression, all statistically significant compared to control. Magnitude of the improvement is not described in the article, only in figures.
	Kim et al., 2019	NOSE, SNOT-22, VAS for nasal obstruction, sneezing, rhinorrhea, snoring, headache, facial pain, and olfactory changes	In ESS group (*n* = 34), xylitol outperformed saline in SNOT-20 improvement (*p* = 0.022). VAS for sneezing, headache, and facial pain favored xylitol (*p* < 0.05). Modified Lund-Kennedy showed no group difference (*p* = 0.466). In CRSsPN, SNOT-20 and VAS scores improved with xylitol (*p* < 0.05). CRSwNP showed no significant difference. Patient preference: More than half preferred xylitol irrigation in both surgical groups.
Manuka Honey	Thamboo et al., 2011	Philpott-Javer Endoscopic Scoring System, SNOT-22, and bacterial culture	Manuka promoted a significant improvement in SNOT-22 scores. No statistically significant change in mucosal edema (PJESS). There was no change in the flora pattern with manuka honey.
	Ooi et al., 2019	LKS, EVA for symptoms, SNOT-22, bacterial culture UPSIT for security	Manuka improved LKS but not SNOT-22 scores. Antibiotic/nasal saline reduced the positivity of the culture compared with manuka/placebo. Manuka/placebo was not superior to the appropriate antibiotic/nasal saline wash.
	Lee et al., 2017	SNOT22, LKS, bacterial culture	There was no difference between Manuka and Saline in terms of: a) Improvement of symptoms, b) Post-treatment culture negativity, and c) Change in the endoscopic Lund-Kennedy score.
	Chang et al., 2011	LKS, EVA merocel discomfort, Histology 7 days after	In the manuka honey group, mixed results on inflammation, no significant difference in mucosal healing. In the gentamicin group, mixed results on inflammation, no significant difference in mucosal healing. Overall, no significant difference in postoperative discomfort and inflammation for manuka honey soaked Merocel compared to Merocel alone.
	Wong et al., 2011	LKS, SNOT-22	In Case 1, SNOT-22 improved significantly with resolved complaints, but some symptoms like nasal irritation emerged. In Case 2, some symptoms improved, but facial pain and nasal obstruction persisted, sinusitis did not improve, and asthma worsened, requiring oral prednisone courses.
Baby Shampoo	Tulaci et al., 2021	LKS, SNOT-22	Baby shampoo promoted better Lund-Kennedy and SNOT-22 scores compared to nasal wash with saline in the 1^st^PO month, but not in subsequent months (3, 6, 12mo). At endoscopy, it promoted improvement in secretion and crusts, but without additional effect on edema and polyp items.
	Chiu et al., 2008	SNOT-22, UPSIT	Baby shampoo improved SNOT-22 scores from 31.6–11.1, especially on the subitems related to posterior rhinorrhea and thick mucus. 63.6 patients showed improvement in UPSIT scores
	Farag et al., 2013	SNOT-22, RSOM-31, PEA test (smell)	Baby shampoo and hypertonic saline improved quality of life (SNOT-22 and RSOM31), with no difference between groups at different endpoints (1–3 weeks, 4–7 weeks, 8–16 weeks).
Colloidal Silver	Scott et al., 2017	SNOT-22, LKS, VAS, microbiology Safety ‒ serum silver dosage, UPSIT, +L10 and VAS	Microbiology result: 18.18% in control group with negative swab and 9.09% in patients with colloidal silver. Visual Analog Scale (VAS) ‒ equal in both groups, without statistical significance. Sino-nasal Outcome Test-22 (SNOT-22): Control without SNOT-22 change, SNOT-22 improvement without colloidal silver, but without statistical significance Lund Kennedy Score (LKS). Improvement in the control and colloidal silver group, but without statistical significance difference.
Povidone-Iodine (PI)	Panchmatia et al., 2019	Modified LKS, culture, SNOT-22	Washing with PVPI promoted after 7 weeks: a) improvement of modified LMS, especially secretion; b) revealing an overall shift from moderate-heavy growth to lighter pathogen growth post PVP-I rinsing. Four of 13 patients depicted no bacterial growth post-PI rinsing; c) Reduction of SNOT22 (33 to 20)
	Wu et al., 2022	SNOT-22, baseline, 1 e 3 meses PO	Both groups showed improvement post-op, however without differences between them (both in terms of endoscopic findings and SNOT-22 scores).
	Lee et al., 2020	Primary- Negative culture Secondary- Tolerability (VAS), SNOT-20, LKS	There were no differences in culture negativity between the 3 groups PI, MUP and saline were well tolerated There was no difference between groups in SNOT-20 and Lund-Kennedy scores
Bacteriophages	Dobretsov et al., 2021	Culture, IL-1b, I-L8, TNF alpha in nasal wash and blood, collected 10 and 30 days after	Otophag reduced S*treptococci* and *Enterobacteriaceae* at 10 and 30 days compared to placebo, with no impact on *Staphylococci*. Otophag reduced CFU numbers. TNF alpha had no reduction. IL-8 reduced in both groups at day 30, with no difference between phage and placebo. IL-1b reduced in phage group at 10 days, while placebo group reduced only on day 30. On day 30, IL-1b was lower in the placebo group than the phage group.
	Rodrigues et al., 2022	Culture, non-standardized endoscopy	After 2 weeks, the patient had significant improvement clinically and at endoscopy. Cultures, for the first time in years, were negative. His right ear also stopped draining. She had a dramatic response, with negative cultures 1 week after starting treatment and a mucosa that looked almost normal. Only subjective parameters analyzed, without quantitative parameters

**Table 3:** Summary of adverse effects reported.

Agent	Authors	Adverse effects
	Rabago et al., 2020	Burning in patients who underwent nasal irrigation (saline and xylitol) but does not report the percentage.
	Silva et al., 2022	Not Informed
Xylitol	Weissman et al., 2011	Light nasal burning in 7% of patients.
	Lin et al., 2017	No adverse effects
	Kim et al., 2019	Not Informed
	Thamboo et al., 2011	Nausea, 3/36 complained of mild burning with nasal spray
	Ooi et al., 2019	No adverse effects
Manuka Honey	Lee et al., 2017	In severe adverse effects. Only 3 who received MH had mild irritation. 100% adherence in both groups.
	Chang et al., 2011	No adverse effects
	Wong et al., 2011	No adverse effects
	Tulaci et al., 2021	Not Informed
Baby Shampoo	Chiu et al., 2008	Nasal irritation, hive
	Farag et al., 2013	52% burning sensation with baby shampoo, 20% discontinue therapy
Colloidal Silver	Scott et al., 2017	Four showed transient elevation of serum silver. No change in UPSIT or major adverse events.
	Panchmatia et al., 2019	Headache, sinus pain, and postnasal discharge
Povidone-Iodine (PI)	Wu et al., 2022	No adverse effects
	Lee et al., 2020	No adverse effects
Bacteriophages	Dobretsov et al., 2021	No adverse effects
	Rodrigues et al., 2022	Not Informed

### Xylitol

Several studies have investigated the effectiveness of xylitol rinsing in the treatment of chronic rhinosinusitis. Four individual randomized controlled trials and one systematic review of randomized controlled trials were found.

Rabago et al.^12^ conducted a study evaluating the effects of xylitol 5% on SNOT-20 and found that the xylitol group showed improvement compared to the control group at 8 and 28 weeks. The saline irrigation group also showed improvement compared to the control at 26 weeks. No significant changes were observed in sleep or wheezing in the active groups, but the xylitol participants reported improvement in sleep compared to baseline.

Silva et al.^13^ examined the impact of xylitol 1.6% on SNOT-22, NOSE score, and VAS. The xylitol group showed greater reductions in SNOT-22, NOSE score, and VAS compared to the saline solution group. Furthermore, within each group, improvements were observed in NOSE scores before and after surgery.

Kim et al.^14^ investigated the effects of xylitol 1.6% in different subgroups. In the ESS group, the xylitol group showed significant improvement in SNOT-20 scores and VAS scores for sneezing, headache, and facial pain. In the CRS without Nasal Polyps (CRSsPN) subgroup, similar improvements were observed, and the xylitol group showed greater improvement in rhinorrhea symptoms among patients with allergic sensitization. Preference surveys indicated that more than half of the patients preferred xylitol irrigation in each surgical group. Overall, nasal irrigation with xylitol was found to be beneficial in postoperative care for ESS and septoplasty, and it provided greater improvement in rhinorrhea symptoms for patients with allergic sensitization compared to saline irrigation.

In a study by Lin et al.,^15^ xylitol 5% improved overall VAS, reduced SNOT-22, increased nasal NO concentration, and right maxillary sinus inducible Nitric Oxide Synthase (iNOS) expression, all statistically significant compared to control (Nasal lavage with saline solution, without xylitol). In another study, Weismann et. al.^16^ showed that xylitol irrigation improved SNOT-20 whereas saline worsened, with xylitol being superior to saline with a statistically significant difference in VAS general symptoms.

The investigations conducted by Weissman et al.^16^ established the comparative advantage of xylitol over saline, while further studies, such as those conducted by Rabago et al.,^12^ Silva et al.,^13^ and Kim et al.,^14^ provided deeper insights into the duration of efficacy, subgroup-specific benefits, and preferences of patients. The studies consistently report that nasal irrigation with xylitol provides significant reductions in symptom scores, particularly SNOT-20 and SNOT-22, as well as enhancements in nasal nitric oxide concentration and related gene expression, compared to placebo. These findings collectively highlight the multifaceted benefits of xylitol in addressing the complex nature of chronic rhinosinusitis.

### Manuka honey

Two randomized clinical trials were conducted to evaluate the effectiveness of manuka honey in treating Chronic Recalcitrant Rhinosinusitis. In one study, by Lee et al.^17^ both the saline and manuka honey (10%) groups showed improvement in SNOT-22 scores after a month of treatment. However, there were no significant differences in other scores like Lund-Kennedy and Lund-MacKay. It is worth noting that the study had limitations as adjuvant systemic therapies were also given. A subgroup analysis suggested that manuka honey may have a chemical microbiological effect and that systemic antibiotics may be more effective when used exclusively with saline irrigation.

In another study, Ooi et al.^18^ compared manuka honey irrigation (16.5%) to saline solution and culture-directed oral antibiotics. The use of culture-directed oral antibiotics and saline rinse appeared to be more effective in eradicating infection in culture compared to manuka honey. However, manuka honey irrigation was well tolerated with no significant changes in symptoms or endoscopic scores.

Overall, these studies suggest that manuka honey irrigations may have a greater impact on microbiological results, particularly in cases of recalcitrant chronic rhinosinusitis. However, there is no evidence of symptomatic or endoscopic benefits compared to saline nasal irrigation. Notably these conclusions are based on a limited number of randomized trials, and larger prospective studies are needed to provide more conclusive recommendations.

In a study by Thamboo et al.^19^ manuka honey (50%) promoted SNOT-22 scores improvement compared to the control group, but there was no significant change in mucosal edema. A double-blinded, randomized, controlled trial by Chang et al.^20^ compared medicated versus unmedicated merocel sponges for functional endoscopic sinus surgery. The study found no statistically significant differences in terms of histopathological inflammation, discomfort during removal, and healing of the postoperative mucosa between drug-soaked merocel and non-medicated merocel. However, the use of budesonide and gentamicin showed a trend towards better healing compared to the control side, while merocel soaked in manuka honey showed no significant difference in healing compared to the control side.

In a case-series study by Wong et al.,^21^ the first patient showed significant improvement in symptoms after 12-weeks of treatment with manuka honey irrigation. However, some post-treatment scores were higher for certain symptoms, suggesting possible nasal irritation. The second patient experienced improvement in some symptoms but continued to complain of facial pain and nasal obstruction, and her sinusitis did not improve while her asthma worsened.

In conclusion, studies on manuka honey in treating Chronic Recalcitrant Rhinosinusitis offer a nuanced view. While suggesting potential microbiological benefits, especially in recalcitrant cases, evidence for symptomatic or endoscopic advantages is inconclusive. Larger trials are needed for definitive recommendations. Varied results highlight the condition's complexity and the need for individualized treatments. Manuka honey's therapeutic potential warrants exploration, but caution is advised due to research limitations and mixed outcomes.

### Baby shampoo

Tulaci et al.^22^ conducted a study comparing the effects of Baby Shampoo Irrigation (BSG) at 1% and Normal Saline Irrigation (NSG) on postoperative outcomes. At the first postoperative month, the BSG group showed significantly better results in terms of postoperative Lund-Kennedy Endoscopic Scores (LKES). However, there were no significant differences between the groups at 3, 6, or 12-months postoperatively. SNOT-22 scores showed a significant reduction in both groups at all time points, but the reduction was significantly better in the BSG group during the first postoperative month. The BSG group also exhibited significant improvements in the rhinological, extranasal, and psychological domains of SNOT-22.

Chiu et al.^23^ conducted in vitro tests and a clinical study to evaluate the effectiveness of baby shampoo irrigation 1%. In the in vitro tests, baby shampoo showed effectiveness in eradicating planktonic *Pseudomonas* at 1% and 10% concentrations and inhibiting biofilm formation. However, it failed to eliminate preformed *Pseudomonas* biofilms. In the clinical study with 18 patients, nasal irrigation with baby shampoo 1% for 4-weeks resulted in subjective improvement in seven patients, as indicated by a reduction in SNOT-22 scores. Most patients reported improvements in discharge thickness and postnasal drainage, and smell test scores also improved in most patients.

Finally, in a study involving 40 adult candidates for endoscopic endonasal surgery with chronic rhinosinusitis by Farag et. al.,^24^ researchers conducted quality-of-life assessments and smell threshold tests before and after surgery. Both baby shampoo 1% and hypertonic saline irrigation groups showed significant improvements in quality-of-life scores, with no significant difference between the two groups. Smell thresholds also improved in both groups. However, the baby shampoo group experienced more side effects and had a higher rate of discontinuation compared to hypertonic saline group, suggesting that tolerability with baby shampoo 1% might be a concern.

### Colloidal silver

A study by Scott et al.^25^ used a total of 22 randomized patients. This study was composed of two groups, both made up of patients with chronic recalcitrant rhinosinusitis. The first group consisted of 10 patients who received only saline solution for nasal wash, while the second group was formed by a group of 12 patients who underwent nasal irrigation with colloidal silver solution (0.015 mg/mL). The result was that there were no statistically significant changes in SNOT-22 or Lund-Kennedy scores over 6-weeks of Colloidal Silver therapy compared to the control. The nasal spray with Colloidal Silver described in the study did not provide subjective or objective improvements among individuals with RSC resistant to conventional clinical treatment. It is suggested that future studies with a larger number of individuals, as well as the use of different devices for nasal irrigation, should be conducted to reach a definitive conclusion.

### Povidone-iodine (PI)

In a study by Lee et al.^26^ the effectiveness of PI solution in nasal irrigations (0.1%) was evaluated. The study compared the culture negativity rates, SNOT-20 scores, and Lund-Kennedy scores among the PI, saline (SAL), and control (MUP) groups. While the PI group had a higher culture negativity rate (70%) compared to the PI (43%) and SAL (47%) groups, statistical analysis did not find significant differences between the treatment groups. Similarly, there were no significant differences in terms of improvement in SNOT-20 and Lund-Kennedy scores among the treatment groups. The statistical analyses did not yield clinically significant results.

In another study,^27^ the efficacy of nasal lavage with PI 0.08% was investigated. The study revealed a significant reduction in the Modified Lund-Kennedy Score after nasal wash with PI. Serum markers of inflammation, including monocytes, neutrophils, IgE, and eosinophils, showed up to a 17% reduction in the PI lavage group. Nasal culture results demonstrated a reduction in bacterial growth after lavage with PI, with four out of thirteen patients showing no bacterial growth. The species identified before treatment included S. aureus (38%), P. aeruginosa (23%), and E. cloacae (15%). The SNOT-22 score also exhibited a significant reduction pre- and post-wash with PI. Two patients experienced subclinical thyroid dysfunction, but no damage to ciliary clearance or olfactory alterations were observed.

Finally, a study by Wu et al.^28^ investigated the efficacy of PI nasal irrigation solution 0.1% postoperatively. The TWSNOT-22 (Taiwan SNOT-22) score and TNR in the PI group showed significant improvement at 1 and 3 months postoperative compared to preoperative measurements. The study also found significant improvements in TWSNOT-22 score, Lund-Kennedy endoscopic score, and TNR results in both groups at 3 months postoperative compared to preoperative measurements. However, there were no significant differences in TWSNOT-22 scores, endoscopic scores, or TNR between the two groups before, 1-month, and 3-months after the operation. All patients had positive bacterial culture results, with no significant differences in bacterial species or growth between the groups. The 0.1% PI nasal irrigation solution was well tolerated by all patients, and no adverse effects were reported during the study.

In summary, studies collectively explore PI nasal irrigation in sinonasal surgery and postoperative care, emphasizing its tolerability and safety without reported adverse effects. Diverse outcomes underscore sinonasal health complexity, requiring further research to define PI irrigation's optimal role in various clinical scenarios. Comprehensive investigations and long-term assessments are needed to fully understand PI irrigation's benefits and limitations in enhancing postoperative outcomes and sinonasal health.

### Bacteriophages

In a randomized, double-blind, placebo-controlled study, by Dobretsov et. al.^29^ bacteriophages were investigated for their potential in treating chronic rhinosinusitis with nasal polyps. The study involved 40 adult patients who underwent functional endoscopic sinus surgery. After surgery, 20 patients received intranasal gel containing a mixture of bacteriophages twice a day for ten weeks, while the other 20 patients received a placebo. The results showed that, on the 10th day of treatment, there was a significant reduction in IL-1β secretion and a decrease in the total number of microorganisms, especially *Enterobacteriaceae,* in the bacteriophage-treated group. *Streptococci* were notably absent in this group as well. Furthermore, the decrease in the activity of secretory IL-1β and IL-8 on the 10th day strongly correlated with the total number of microorganisms. On the 30th day, the decrease in serum IL-1β significantly correlated with the total number of microorganisms and enterobacteria in the bacteriophage-treated group. These findings suggested that bacteriophage administration restored the microbial balance in the nasal cavity and reduced the inflammatory response in chronic rhinosinusitis with nasal polyps. These changes, particularly the reduction in inflammation, may potentially help prevent the recurrent growth of polyp tissue in the future.

A case report by Rodriguez et al.^30^ has shown that after 2 weeks of bacteriophage treatment, the patient had significant improvement clinically and at endoscopy. Cultures, for the first time in years, were negative. The right middle ear also stopped draining. One week after bacteriophage treatment, the sinonasal mucosa looked almost normal.

In summary, Dobretsov et al.^29^ studied bacteriophages as a treatment for chronic rhinosinusitis with nasal polyps. In a 40-patient trial, intranasal bacteriophages led to reduced IL-1β secretion, lower microorganisms and absent Streptococci after 10 days. Correlations between decreased inflammatory markers and microbial balance on days 10 and 30 suggested a positive impact. The study suggested bacteriophages restored microbial balance, reducing inflammation and potentially preventing recurrent polyp tissue growth. Rodriguez et al.^30^ case report supported these findings, showing clinical improvement, negative cultures after years, cessation of middle ear drainage, and near-normal sinonasal mucosa within two weeks of bacteriophage treatment.

## Discussion

The pathogenesis of CRS is widely recognized to involve multiple etiologies and modifying factors that contribute to the final state of chronic inflammation of the nose and paranasal sinuses. In some subtypes of CRS, bacterial colonization certainly plays a major role in inducing or modifying the inflammatory response by the host. Notably, recent microbiome studies have revealed that the sinonasal cavity is colonized by a high number of microbial species, most of them not detected by conventional cultures.^31^, ^32^ The presence of dysbiotic flora, which includes an unbalanced abundance of pathogenic and commensal microbiota, as well as pathogenic biofilm colonization of the sinonasal cavity, are potential therapeutic targets that need to be addressed to control inflammation in CRS patients.

Managing bacterial infections and biofilm eradication in CRS is challenging due to antibiotic resistance and side effects. To address this, non-antibiotic antimicrobial agents have emerged for CRS, aiming to improve patient outcomes by reducing bacterial colonization.

One of the products that has been widely tested is xylitol. Xylitol is a naturally occurring pentose sugar with multiple antibacterial properties, including inhibition of growth and adhesiveness of pathogenic bacteria, and enhancement of the innate immune system by decreasing salt concentration of the airway surface.^33^ This natural sugar may have applications in preventing tooth decay^34^ and managing acute ear infections^35^ and post-operative cases of mixed CRS phenotypes/endotypes. Additionally, limited trials have explored its suitability for addressing chronic upper respiratory symptoms. The collective body of evidence from multiple studies supports the efficacy of xylitol rinsing as a promising therapeutic approach for the management of chronic rhinosinusitis. The current evidence consistently demonstrates the superior effectiveness of xylitol over saline irrigation in alleviating sinusitis symptoms, as measured by various standardized assessment tools including the Sinonasal Outcome Test (SNOT) and Visual Analog Scale (VAS). Xylitol not only reduces symptom severity but also contributes to overall improvements in patients' quality of life. Overall, xylitol has been shown to reduce inflammation and prevent bacterial growth, which can help alleviate CRS symptoms. It is noteworthy that xylitol's positive impact is not limited to a specific subset of patients. Xylitol irrigation offers tailored treatment across diverse profiles and postoperative situations. The research highlights its potential as an adjunctive therapy in chronic rhinosinusitis management, potentially improving outcomes compared to saline. Further research and clinical validation are needed to establish its role in chronic rhinosinusitis treatment fully.

Manuka honey is another complex compound that has been used elsewhere to treat injuries that are prone to bacterial biofilm formation. There are several properties that are believed to contribute to its effectiveness, such as changes in pH, increased osmolarity, and the presence of unique compounds like methylglyoxal,^36^ which is effective against bacteria and biofilms like *P aeruginosa* and *S aureus*.^37^ Some trials testing manuka honey in CRS patients suggest that nasal irrigations with manuka honey may have a greater impact on microbiological results, particularly in reducing pathogenic flora in cases of recalcitrant chronic rhinosinusitis. Overall, the studies on manuka honey irrigation in chronic rhinosinusitis suggest potential microbiological benefits but lack evidence of significant symptomatic or endoscopic improvements compared to saline irrigation. It is important to note that some studies used appropriate oral antibiotic therapy on both arms (manuka honey and saline), which might have decreased potential benefits of manuka irrigations. Larger studies are needed to provide more conclusive evidence and recommendations on nasal irrigations using manuka honey.

Surfactants are a well-known group of antimicrobial agents widely used in skin care. In this sense, diluted concentrations of baby shampoo as a nasal rinse have been proposed as a treatment option for CRS, due to its putative ability to break down and emulsify mucus and other debris in the sinuses, and eventually improving mucus clearance.^23^ In addition, baby shampoo may also present anti-inflammatory and antimicrobial effects in the nose. Some studies have shown that the ingredients in baby shampoo, such as tea tree oil and aloe vera, have anti-inflammatory properties that can reduce the inflammation in the sinuses, as well as antimicrobial properties, reducing the growth of bacteria and fungi, as well as removing mature biofilms.^23^ The use of dilute baby shampoo for nasal irrigation has shown positive effects, including increased nasal mucociliary clearance time and improved postoperative outcomes in terms of endoscopic scores. It has also led to subjective improvements in symptoms and smell test results in patients with chronic rhinosinusitis. However, nasal irritation and therapy discontinuity may limit adherence. More extensive research on efficacy, safety, and long-term effects is needed.

Silver is a chemical element that exhibits activity against Gram-positive and Gram-negative microorganisms, fungi, protozoa, and even some viruses. In recent times of increasing numbers of bacteria resistant to antibiotics, silver has emerged as a possible option for the treatment of chronic infections.^38^ Colloidal silver, an aqueous suspension with silver particles, has been studied as an alternative for topical treatment in patients with Chronic Recalcitrant Rhinosinusitis. Despite promising antibacterial results in vitro, colloidal silver irrigation did not provide subjective or objective improvements among individuals with recalcitrant chronic rhinosinusitis in a small sample size study. Further studies are still warranted for a definitive conclusion.

Povidone-Iodine (PI) is an antiseptic agent that has been used for the treatment of various infections, including CRS. The mechanism of action of PI for the treatment of CRS is not fully understood. One proposed mechanism is that PI releases free iodine, which oxidizes key microbial components such as proteins, fatty acids, and nucleotides, and leads to cell death of a broad variety of microorganisms, including those that are resistant to antibiotics.^39^ In addition to its direct antimicrobial effects, PI has been shown to have anti-inflammatory properties, reducing the production of proinflammatory cytokines. Overall, the mechanisms of action of PI for the treatment of CRS are likely to involve both direct antimicrobial effects and anti-inflammatory effects.^40^ Despite its promising potential effects, the two controlled trials that investigated the effects of PI on CRS patients showed no differences in terms of SNOT-22 improvement or negativity in cultures relative to controls. The determination of the optimal dose of PI as well as further studies involving a larger number of individuals are still required.

Bacteriophages are bacteria-specific viruses used as a potential treatment for CRS. They target bacteria involved in sinonasal infections by attaching to bacterial cell receptors, injecting genetic material and disrupting normal processes, causing bacterial death. Moreover, bacteriophages might stimulate the immune system, producing cytokines that fight infection and reduce inflammation. Their action in CRS likely involves both direct bactericidal and immunomodulatory effects. While one experimental study showed promising results with reduced bacteria, data on efficacy and safety are limited and require further exploration.^41^

In summary, most alternative antimicrobials for CRS lack extensive clinical trials. Patients generally tolerated tested doses, except for baby shampoo 1%, with some reporting mild nasal discomfort. Heterogeneity among studies, encompassing various CRS types, surgical status, timeframes, and bacterial infection association, limits conclusive findings. Nevertheless, some agents show promise as antimicrobial and anti-inflammatory treatments and need larger, condition-specific controlled trials for further exploration.

## Conclusion

In conclusion, non-antibiotic treatments such as xylitol, manuka honey, baby shampoo, colloidal silver, Povidone-Iodine (PI) and bacteriophage therapy have shown potential in the management of CRS in adult patients. Among these non-antibiotic agents, xylitol has the best level of evidence in providing benefits to CRS patients, reducing sinonasal symptoms, especially in the post-operative period. Manuka honey and baby shampoo are potential antibacterial agents in vitro, nonetheless, there is no evidence whether their use in nasal irrigations adds benefits in comparison with controls. Colloidal silver, PI, and bacteriophages topically applied in the nose may reduce bacterial colonization, however with no clear evidence of whether these agents provide improvement in sinonasal symptoms or benefits in modifying nasal endoscopic scores. Further studies are still needed for more definitive conclusions.

## Funding

This study was financed in part by the Coordenação de Aperfeiçoamento de Pessoal de Nível Superior ‒ Brasil (10.13039/501100002322CAPES) ‒ Finance Code 001.

## Conflicts of interest

The authors declare no conflicts of interest.
